# Root canal adaptation and intra-tubular penetration of three fiber-post cementation systems

**DOI:** 10.4317/jced.55208

**Published:** 2018-12-01

**Authors:** Carmen Llena, María García -Gallart, Leopoldo Forner, Marco Ferrari

**Affiliations:** 1 MD, DDS, PhD. Department of Stomatology. Universitat de València (Spain). C. Gascó Oliag, 1. 46010 Valencia (Spain); 2 DDS. Department of Stomatology. Universitat de València (Spain). C. Gascó Oliag, 1. 46010 Valencia (Spain); 3 Department of Dental Materials and Fixed Prosthodontics. University of Siena (Italy). Policlinico Le Scotte, Viale Bracci. 53100 Siena (Italy)

## Abstract

**Background:**

To measure the penetration of three bonding systems for the luting of fiber glass posts in endodontically treated teeth, using confocal laser scanning microscopy (CLSM).

**Material and Methods:**

A total of 30 maxillary incisors were shaped with the Mtwo system and filled with gutta-percha and Top Seal cement. The sample was divided into three groups (ni=10) according to the bonding system used to cement the posts: Group 1 (Prime&Bond NT and Rebilda DC with a total-etch technique); Group 2 (Futurabond DC and Rebilda DC with a dentin self-etch technique); and Group 3 (BisCem self-adhesive cement). Rhodamine B was added to the bonding systems to allow visualization by CLSM. Three 1-mm thick cross-sections were obtained of each root at a distance of 2, 5 and 8 mm from the coronal limit of the root. The specific software of the CLSM system was used to measure the percentage of the root canal perimeter showing penetration of the bonding system in the dentinal tubules, together with the maximum depth of penetration. Comparison between groups were made by Kruskal Wallis test, and comparison two by two groups with Mann-Whitney U-test.

**Results:**

Depth of penetration of the resin tags, were distributed from greater to lesser depth as follows: BisCem > Prime&Bond NT > Futurabond DC. BisCem showed significantly greater penetration in the middle and apical thirds than the rest of the systems (906.14±67.42 and 699.27±76.26 µm, respectively). The percentage perimeter exhibiting penetration in the coronal third was significantly greater with BisCem versus Futurabond DC (56.08±7.24 and 44.38±5.23%, respectively). No significant differences were recorded in the middle and apical thirds among the three systems.

**Conclusions:**

BisCem resulted in greater depth of intratubular penetration at all studied levels. The percentage perimeter of the canal showing penetration was similar for all the bonding systems.

** Key words:**Fiber post luting, sealer adaptation, confocal laser scanning microscope.

## Introduction

The reconstruction of a tooth after root canal treatment is complicated when the crown is partially or completely destroyed. As a result, it is sometimes necessary to use intra-root components to afford greater retention of the restoration (1).

The use of the fiber glass reinforced posts has increased in recent years compared with the use of posts made of other materials, since the former offer a number of advantages: an elastic modulus similar to that of dentin, thereby allowing uniform distribution of tension along the longitudinal axis of the root (2); good esthetics in anterior sectors; and translucency allowing light transmission for correct polymerization of the bonding system (3,4)

Post retention requires the use of bonding systems and cements based on resins. At present, we can use total-etch and self-etch bonding systems that are combined with a cement or, alternatively, self-adhesive resin cement (SARC) can be employed. The development of new materials and techniques has made it possible to reduce the number of clinical steps and the accumulation of possible errors in the different stages of post cementing (5).

The total-etch bonding systems require dentin preparation with phosphoric acid and placement of the adhesive in the canal walls before inserting the cement and post. The complexity of the procedure, the access difficulties, and the numerous steps involved may be a cause of failure (6).

The self-etch bonding systems use a functional monomer that penetrates the dentinal tubules together with the resin monomers without the need for prior elimination of the smear layer. The underlying adhesion mechanism combines micromechanical and chemical actions, characterized by interaction between the acid monomers and hydroxyapatite (7). When these systems are used for post cementing, adhesion is fundamentally attributable to chemical interaction between the acid monomers and hydroxyapatite, due to the potential presence of remaining dentin, gutta-percha and endodontic cement within the canal (8,9).

In addition to insertion in a single clinical step, the recently introduced SARC offer the advantage of requiring no previous dentin treatment. This simplification of the technique could help avoid the accumulation of errors in preparing the canal. Such cements are composed of acid monomers capable of demineralizing and infiltrating the dentin even in the presence of the smear layer. The adhesion mechanism is therefore based on micromechanical binding and chemical interaction between the cement and hydroxyapatite (10).

The adhesive processes in the insertion of posts have a number of specific characteristics. On one hand, the dentin is dehydrated, and on the other the debris generated during preparation of the canal for placement of the post contain not only dentin particles but also remaining traces of gutta-percha and sealer cement (11,12). The presence of all these elements can directly affect preparation of the dentin, its infiltration by the adhesive, and retention of the post (13)

Many studies have used push-out techniques to evaluate the adhesion strength of different cementing procedures, different types of posts, treatments before post placement, canal preparation, etc. However, little information is available on the adaptation of the bonding systems and cement to the walls of the root canal and their penetration of the dentinal tubules in teeth subjected to previous canal treatment. The present study was therefore carried out with the following objectives: to analyze the depth of penetration in the dentinal tubules and the percentage perimeter with penetration of three bonding systems for the cementing of posts (a total-etch bonding system with conventional cement, a self-etch bonding system with conventional cement, and a SARC), using confocal laser scanning microscopy (CLSM). In all cases the adhesive systems were applied following canal preparation and filling with gutta-percha and sealer cement. The following null hypotheses were tested: H01 = no differences in tubular penetration among the three bonding systems; and H02 = no differences in the perimeter of the canal showing penetration among the three bonding systems used to cement the post.

## Material and Methods

The present study was approved by the Ethics Committee of the University of Valencia -Valencia, Spain- (Reference: H1459370276741).

-Preparation of the samples

We selected 30 human maxillary incisors extracted due to periodontal reasons, without coronal caries, with a sealed apex and without root resorptions, pulp calcifications or other morphological anomalies. Two X-ray projections were used to confirm the presence of a single straight canal. The teeth were stored in 0.1% thymol solution until processing.

The teeth were sectioned below the cement-enamel junction using a tungsten carbide disc (Komet Dental, Lemgo, Germany) operated at low speed with refrigeration, in order to obtain working specimens measuring 16 mm in length, which were stored in saline solution during the study. The access cavity was prepared with a size 016 round diamond drill (Komet Dental, Lemgo, Germany). For the canal measurements, we inserted a K10 file (Dentsply/Maillefer, Ballaigues, Switzerland), advancing it until the tip was visualized through the apex. The length was measured, and 0.5 mm was subtracted to yield the working length for the rest of the instruments used.

All the canals were prepared with the Mtwo system (15.05, 20.06, 25.06; VDW, Munich, Germany). Irrigation during instrumentation was carried out using 5.25% sodium hypochlorite. As final irrigation we applied 2 ml of 5.25% sodium hypochlorite, followed by 2 ml of physiological saline, 1 ml of 17% EDTA during one minute, 2 ml of physiological saline and 2 ml of 5.25% sodium hypochlorite, in succession. All specimens were dried with sterile paper tips before filling (VDW, Munich, Germany).

Filling was performed using the lateral condensation technique with Top Seal cement (Dentsply Maillefer, Konstanz, Germany). The openings were sealed with composite, and the specimens were stored in physiological saline until processing.

-Placement of the post

One week after canal treatment, a fiber glass post was inserted in each canal, randomizing the teeth to three groups (ni=10) according to the bonding system used.

The coronal gutta-percha was partially eliminated using a Peeso drill (VOCO, Cuxhaven, Germany) measuring 0.7 mm in diameter. Space was then created for placement of the post using the drill of the Rebilda Post system (VOCO, Cuxhaven, Germany), measuring 1.2 mm in diameter. We left 6 mm of gutta-percha in the apical third of each root. Irrigation was carried out in the following sequence: 2 ml of 5.25% sodium hypochlorite, 2 ml of physiological saline, 1 ml of 17% EDTA during one minute, and 2 ml of distilled water.

Rebilda Post fiber glass posts (VOCO, Cuxhaven, Germany) were cemented, without previous treatment of the post surface, following the specific procedure for each group.

Group 1: Etching with 37% orthophosphoric acid during 15 seconds, washing with water for 15 seconds, drying with paper points, placement of Prime&Bond NT (Dentsply, Milford, USA) with Endo Tim (VOCO, Cuxhaven, Germany), application of air during 5 seconds, placement of Rebilda DC cement (VOCO, Cuxhaven, Germany) with an applicator, insertion of the post and polymerization during 40 seconds.

Group 2: Drying of the interior of the canal with paper points, placement of Futurabond DC self-etch adhesive (VOCO, Cuxhaven, Germany) with Endo Tim (VOCO, Cuxhaven, Germany), application of air during 5 seconds, placement of Rebilda DC cement (VOCO, Cuxhaven, Germany) with an applicator, insertion of the post and polymerization during 40 seconds.

Group 3: Drying the canal with paper points, placement of BisCem self-adhesive cement (Bisco, Schaumburg, USA) within the canal using an applicator, insertion of the post and polymerization during 40 seconds.

In order to visualize penetration of the bonding system used to cement the post in the interior of the dentinal tubules, we mixed the Prime&Bond NT, Futurabond DC and BisCem with 1% Rhodamine B (Sigma-Aldrich, Steinheim, Germany).

The roots with the posts were stored in physiological saline for 7 days, after which three 1-mm thick cross-sections were obtained of each root at a distance of 2, 5 and 8 mm from the coronal limit of the root.

-Confocal laser scanning microscopy

The root sections were polished with Sof-Lex discs (3M, Austin, USA) of decreasing particle size, followed by examination with a confocal laser scanning microscope -CLSM- (Confocal Spectral Leica SP2, Leica Microsystems, Heidelberg, Germany) under x40 magnification. The penetration of the Rhodamine B within the dentinal tubules was measured in µm using the software of the microscope system (LCS Lite v. 2611537 Leica, Heidelberg, Germany). We also recorded the percentage of the root canal perimeter showing tubular penetration in each section. All the observations were made by the same examiner, and 10% of the measurements were randomly repeated on a blind basis to establish consistency.

-Statistical analysis

The data were processed using the SPSS® version 21.0 statistical package (SPSS Inc.; Chicago, IL, USA). After confirming that the two variables (penetration in µm and percentage perimeter with penetration) not exhibited a normal distribution within each group, nonparametric test were applied. To compare penetration depth and perimeter with penetration between groups Kruskal Wallis test was used, for comparison groups two by two Mann-Whitney U-test was used. The Friedman test in turn was used to compare the different levels of the sections (coronal, middle and apical third).

## Results

**Figure 1:**
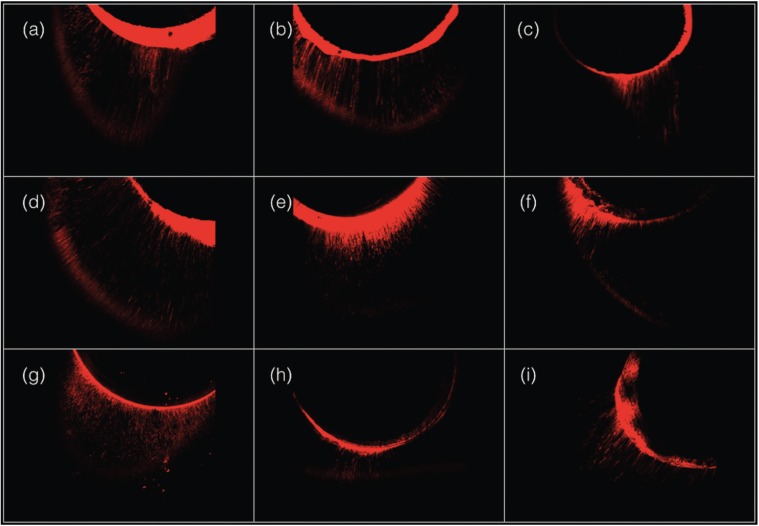
Maximum tubular penetration of the bonding systems. BisCem group: (a) coronal third; (b) middle third; (c) apical third. Prime&Bond NT / Rebilda DC group: (d) coronal third (e) middle third; (f) apical third. Futurabond / Rebilda DC group: (g) coronal third; (h) middle third; (i) apical third.

The mean tubule penetration values of the bonding systems are reported in  Table 1. Greater penetration was observed with the BisCem system in all three tooth sections. In the coronal third, significant differences were observed between BisCem and Futurabond (915.84 ± 44.78 versus 701.89 ± 41.76 µm, respectively) and between Prime&Bond NT and Futurabond (880.24 ± 66.69 versus 701.89 ± 41.76 µm, respectively). In the middle and apical thirds, significant differences were recorded among the three cementing systems (*p*<0.05) - BisCem being the system with the greatest penetration capacity, followed by Prime&Bond NT and Futurabond. The depth of penetration decreased significantly in all the groups from coronal to apical. Figure 1 shows the depth of penetration of the bonding system in the three groups, identified by means of the Rhodamine B.

**Table 1:**
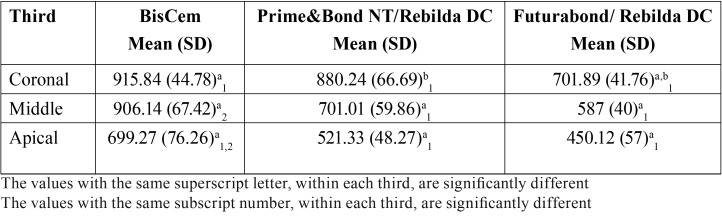
Depth of penetration (µm) of the bonding systems within the dentinal tubules.

**Figure 2:**
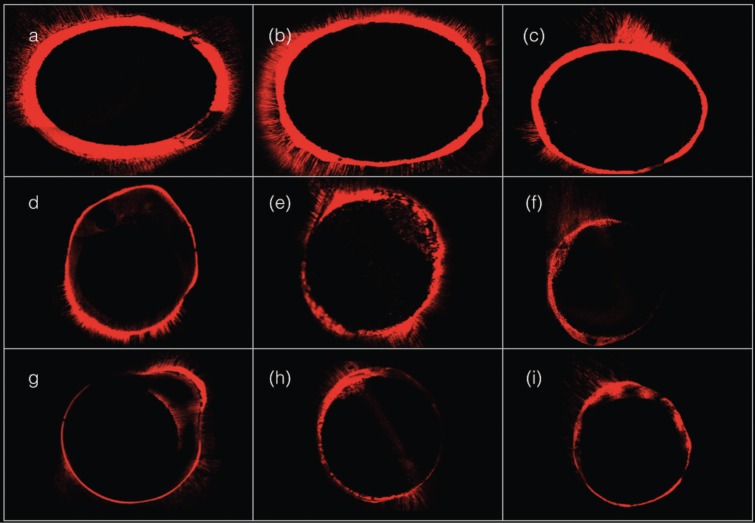
Perimeter of the canal with tubular penetration of the bonding systems. BisCem group: (a) coronal third; (b) middle third; (c) apical third. Prime&Bond NT / Rebilda DC group: (d) coronal third (e) middle third; (f) apical third. Futurabond / Rebilda DC group: (g) coronal third; (h) middle third; (i) apical third.

Regarding the percentage perimeter with penetration,  Table 2 shows a significantly greater percentage in the BisCem group versus Futurabond in the coronal third (56.08 ± 9.29 versus 44.38 ± 5.23%, respectively). In the middle and apical thirds, no significant differences were recorded among the three groups, though slightly higher values were obtained with Futurabond. Figure 2 shows the perimeter with penetration of the bonding system in the three groups, identified by means of the Rhodamine B.

**Table 2:**
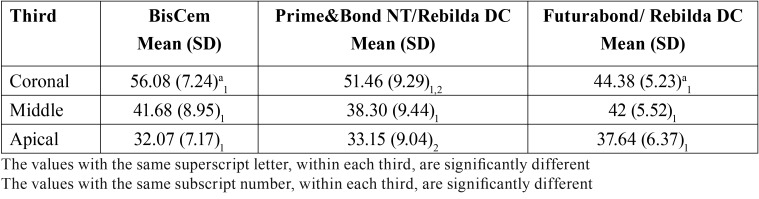
Percentage perimeter of the root canal showing penetration for each bonding system.

## Discussion

The first null hypothesis was rejected, since the BisCem cement system showed significantly greater penetration depths than the other two systems. The second null hypothesis was also rejected, since significant differences in percentage perimeter with penetration between BisCem and Futurabond were obtained in the coronal third.

After preparation of the root canal for post placement, the dentinal smear layer formed dur-ing preparation must be eliminated in order to facilitate intratubular penetration of the bonding sys-tem, since failure doing so would cause post retention to depend mainly on friction (14-16), for this reason we performed irrigation with sodium hypochlorite and EDTA, followed by a final irrigation with distilled water. The excess of water was removed with paper points in order to preserve dentin humidity. Any surface treatment of the post were performed, according the results of Rathke *et al.* (14)

Irrigation with EDTA before using the BisCem is controversial, since it may negatively affect adhesion of the latter (15). However, Faria-e-Silva *et al.* in 2013 (16) demonstrated that irrigation with EDTA before placing the BisCem increases bonding strength. We therefore decided to use the same pre-post cementing irrigation protocol in all three groups.

A microbrush was used to apply the bonding system within the canal, since a number of authors have shown the microbrush to allow more uniform application along the full length of the root and significantly improve access to the apical third (17).

The depth of tubular penetration and the percentage of canal perimeter showing penetration have been studied using confocal laser scanning microscopy (CLSM), in accordance with the method proposed by other authors (18,19). Thanks to the use of Rhodamine B, this technique allows great precision in assessing the depth and number of tubules showing penetration, as well as the perimeter of the canal in which the marked material is located. Bitter *et al.* in 2009 (18) and Marigo *et al.* in 2012 (20) demonstrated that fluorochrome binding with the cements or adhesive-systems to which they are added does not modify the properties of the latter (20,21). With this examination process it is not necessary to subject the samples to any drying processes (as occurs with scanning electron microscopy, for example) that might result in material disadaptation or scaling.

In this study the goal was to evaluate penetration deep and percentage of canal penetration by the adhesive system. This was the reason that Rhodamine B was only placed in the adhesive system when the dual cement was used in combination with a self-adhesive or total-etch adhesive system for fixing the post. In the SARC group no other adhesive-system was used, and Rhodamine B was mixed with the cement.

Bonding of the adhesive to the dentin is conditioned by a number of variables, including the tubular structure, organic contents and moisture. After canal treatment, the dentin is dehydrated and is a deficient tissue for the application of bonding systems such as Prime&Bond NT, which uses acetone as vehicle (22). Nevertheless, we used it in representation of a total-etch system in our study because it is frequently used in clinical practice. We decided to apply the product in a single layer (23) with drying during 5 seconds, since prolongation of the drying time results in thinning of the layer (24), which could adversely affect bonding performance.

There is controversy regarding the bonding of self-etch adhesives with dual polymerization resin cements, since the acid monomers of the adhesive could interfere with setting of the cement consuming the tertiary amines required for starting polymerization (6). On the other hand, these adhesives behave as permeable membranes after polymerization, allowing the passage of fluids and affecting bonding between the adhesive and the cement (13). In our study we decided to evaluate bonding between the Futurabond self-etch adhesive and Rebilda DC dual cement, since these two products are marketed for combined use.

Some mechanical traction studies have reported low resistances when combining self-etch bonding systems and dual polymerization cements (25). Our own findings confirm poorer performance on combining the Futurabond self-etch adhesive with Rebilda DC resin cement.

Regarding SARC, no relationship has been described between the depth of penetration of the cement and the strength of bonding of glass fiber posts to the dentin (18). Previous etching of the dentin results in slightly lesser bonding resistance of BisCem (26). In our study we decided not to etch the dentin before placement of the cement, in concordance with the recommendations of the manufacturer, and in all thirds we observed significantly greater penetration depths than with the other bonding systems tested.

Different authors have studied post adhesion to root dentin using push-out tests (27,28). The use of BisCem and RelyX U100 with fiber posts has resulted in greater bonding strength compared with RelyX ARC dual cement (29). In another study (27) likewise comparing SARC and dual cements in application to post placement, the authors concluded that the former afford greater bonding strength, particularly in the apical third of the canal, while bonding strength in the case of dual cements proved more homogeneous along the length of the canal. Similar conclusions were drawn on comparing the bonding resistance of two SARC (BisCem and RelyX Unicem) versus two dual cements (Panavia-F and Esthetic) in application to fiber posts (29).

In a previous study carried out by our group, BisCem showed the greater cement penetration and percentage of the perimeter with penetration in all root thirds, with respect to Prime&Bond NT combined with Rebilda DC using a total dentin etching technique (30). In this study, we found that BisCem shows a significantly greater percentage of perimeter with penetration than the self-etch adhesive and dual cement group, in the coronal third. However, in the rest of the canal, all the tested systems showed similar percentage penetration values. With regard to deep penetration, BisCem showed the greatest values in all thirds. In principle, a greater percentage of perimeter with penetration and a greater penetration deep should result in greater adhesion strength.

Coronal to apical reduction in the depth of penetration and in the percentage of tubular perimeter with penetration has been reported in the literature, and was confirmed in our study in all the groups. This phenomenon could be attributed to reduction of the dentinal tubules from the coronal third of the root canal to the apical third, greater apical sclerosis, and difficulties in positioning the materials despite the use of microbrushes.

The results obtained in our study and the data found in the literature recommend the use of SARC for the insertion of fiber glass posts in root canals, in view of the good performance at the cement material – root dentin interface, and the simplicity of the procedure.

## Conclusions

In conclusion, from the study of the different post cementing systems analyzed, BisCem resulted in greater depths of intratubular penetration at all studied levels. The percentage perimeter of the canal showing penetration was similar for all the bonding systems, and only the BisCem system showed higher values in the coronal third versus Futurabond / Rebilda DC.
